# Temporal dynamics of liver mitochondrial protein acetylation and succinylation and metabolites due to high fat diet and/or excess glucose or fructose

**DOI:** 10.1371/journal.pone.0208973

**Published:** 2018-12-26

**Authors:** Jesse G. Meyer, Samir Softic, Nathan Basisty, Matthew J. Rardin, Eric Verdin, Bradford W. Gibson, Olga Ilkayeva, Christopher B. Newgard, C. Ronald Kahn, Birgit Schilling

**Affiliations:** 1 Buck Institute for Research on Aging, Novato, California, United States of America; 2 Joslin Diabetes Center, Department of Medicine, Harvard Medical School, Boston, Massachusetts, United States of America; 3 Boston Children’s Hospital, Division of Gastroenterology, Boston, Massachusetts, United States of America; 4 Discovery Attribute Sciences, Therapeutic Discovery, Amgen, South San Francisco, California, United States of America; 5 Sarah W. Stedman Nutrition and Metabolism Center and Duke Molecular Physiology Institute, Duke University Medical Center, Durham, North Carolina, United States of America; 6 Department of Pharmacology and Cancer Biology and Department of Medicine, Duke University Medical Center, Durham, North Carolina, United States of America; 7 Department of Medicine, Division of Endocrinology, Metabolism, and Nutrition, Duke University Medical Center, Durham, North Carolina, United States of America; East Tennessee State University, UNITED STATES

## Abstract

Dietary macronutrient composition alters metabolism through several mechanisms, including post-translational modification (PTM) of proteins. To connect diet and molecular changes, here we performed short- and long-term feeding of mice with standard chow diet (SCD) and high-fat diet (HFD), with or without glucose or fructose supplementation, and quantified liver metabolites, 861 proteins, and 1,815 protein level-corrected mitochondrial acetylation and succinylation sites. Nearly half the acylation sites were altered by at least one diet; nutrient-specific changes in protein acylation sometimes encompass entire pathways. Although acetyl-CoA is an intermediate in both sugar and fat metabolism, acetyl-CoA had a dichotomous fate depending on its source; chronic feeding of dietary sugars induced protein hyperacetylation, whereas the same duration of HFD did not. Instead, HFD resulted in citrate accumulation, anaplerotic metabolism of amino acids, and protein hypo-succinylation. Together, our results demonstrate novel connections between dietary macronutrients, protein post-translational modifications, and regulation of fuel selection in liver.

## Introduction

Lysine residues in proteins are a point of convergence for multiple PTMs, especially acylation. Lysine acetylation was first reported in nuclear proteins [1,2]. More recently, immuno-affinity enrichment and mass spectrometry were used to discover that acetylation is also highly abundant in mitochondrial proteins [3]. Modification of protein lysine residues by other acyl groups was discovered later, including succinylation [4]. The diversity of lysine acylations is mirrored by the diversity of reactive acyl-CoA species (RACS), and includes malonylation and glutarylation [5–7]. Similar to acetylation, succinylation extensively modifies mitochondrial proteins [8], but is also found in other cellular compartments [9]. Protein acetylation and succinylation are reversed by sirtuins, an NAD+-dependent class of lysine deacetylases [10]. In the mitochondria, Sirt3 and Sirt5 efficiently remove acetyl and succinyl groups, respectively [8,11–14]. Reversible modification of mitochondrial proteins by acylation may allow tuning of mitochondrial function in response to nutrient availability, which costs less energy than degradation and synthesis of new proteins. Although there is a clear correlation between protein acylation and various phenotypes, the underlying causal relationship between phenotype, protein function, and protein acylation is still poorly understood.

Due to the prevalence of acetylation and succinylation on metabolic enzymes in the mitochondria, and the lack of evidence for a mitochondria-targeted acyl transferase, the growing consensus is that mitochondrial protein acylation is a non-enzymatic consequence of metabolism [15]. Such non-enzymatic modification may be facilitated by a combination of high pH in the mitochondrial matrix, altered lysine side-chain pKa, the composition of nearby amino acids, and changes in levels of RACS under different metabolic conditions [16,17]. RACS composed of CoA and dicarboxylic acids appear to have a unique reactivity due to intramolecular base catalysis that can spontaneously form high-reactivity anhydrides *in vitro* and *in vivo* [18]. The non-enzymatic modification model is further supported by the correlation of RACS concentrations with the prevalence of specific RACS modifications, as well as the sirtuin deacetylase enzymes that remove them, across various tissues [19]. Therefore, alteration of protein acylation should be interpreted in the context of RACS abundance and metabolism.

Mitochondrial protein hyper-acetylation has been associated with changes in protein function and with various genotypes and metabolic stressors, including Sirt3 knockout [20], ethanol exposure [21], chronic caloric restriction [22], ketogenic diet [23], or chronic HFD [24]. HFD-induced mitochondrial protein hyper-acetylation has been studied extensively, and one study suggested protein acetylation is solely driven by acetyl-CoA produced from fatty acid oxidation [25]. Notably, mice fed HFD for 13 weeks, or Sirt3 KO mice, show accelerated development of metabolic disorders and liver inflammation [26]. Compared to the number of studies that demonstrate hyper-acetylation, only a few conditions are associated with protein hyper-succinylation, such as mutations in isocitrate dehydrogenase associated with cancer [27], succinyl-CoA ligase deficiency [9], and knockdown of Sirt5 [8,13].

Several reports describe how site-specific protein acylation can alter functions of proteins in multiple pathways. Similar to other protein modifications such as phosphorylation, protein acylation has been reported to cause both enzyme activation and inhibition. For example, carbamoyl phosphate synthase-1 (Cps1), which is required for ammonia clearance and amino acid catabolism, is deacetylated by Sirt5 to cause increased activity, thereby enabling the shift to amino acid catabolism during fasting [28]. Ketone body production is decreased in the absence of Sirt5, and an enzyme required for ketone body production, HMG CoA synthase 2 (Hmgcs2), appears to be inhibited by succinylation at K83 and K310 [8]. In addition, hydroxyacyl CoA dehydrogenase (Hadha), a subunit of the trifunctional enzyme required for beta-oxidation of fatty acids, is inhibited by succinylation of K351 [19], and long-chain acyl-CoA dehydrogenase (LCAD) activity is inhibited by acetylation of K318 and K322 [29]. Although less common, there are reports of functional activation of proteins by acylation. For example, the transcription factor ChREBP shows enhanced activity when acetylated on K672 [30]. Thus, system-wide analysis of changes in protein acylation patterns, instead of effects on individual proteins, may be a roadmap for better understanding of regulatory mechanisms that drive physiologic phenotypes.

Although there are examples of acylation influencing metabolism, it is still unclear how diet composition can influence protein acylation. Since multiple acyl groups can modify any given lysine, studies that simultaneously assess the dynamic interplay between these modifications are needed to provide a more complete understanding of acylation and regulation of protein activity [31]. Here, we fed mice SCD or HFD with or without supplemented fructose or glucose, and quantified liver mitochondrial proteins, mitochondrial protein acetylation and succinylation, as well as hepatic organic acids, acyl CoA, acylcarnitine, and amino acids levels. To rapidly and comprehensively quantify acetylation and succinylation, we combined immunoprecipitation, quantitative, label-free, data-independent acquisition (DIA) mass spectrometry [32,33], and automated data analysis with the PTM identification and quantification from exclusively DIA (PIQED) workflow [34]. This multi-omics approach, which combines quantitative analysis of proteins, PTMs, and metabolites, provides a comprehensive view of molecular responses to chronic changes in diet composition, and resultant changes in macronutrient signaling and selection in the liver. We find a dichotomous response to excess dietary sugar and fat; feeding excess glucose for 10 weeks (and to a lesser extent fructose) induces mitochondrial protein hyper-acetylation, but HFD alone does not. Instead, chronic HFD caused protein hypo-succinylation and increased citrate, a known inhibitor of glycolysis.

## Materials and methods

### Experimental design and description of samples

In this study there were 6 distinct diet groups collected after 2 weeks or 10 weeks of feeding, resulting in a total of 12 groups. Each group had 5 biological replicates, resulting in a total of 60 mice used for this study. From those 60 mice we analyzed 240 samples. From 60 liver mitochondria samples, we generated 60 samples for quantification of proteins, 60 samples for quantification of protein acetylation, and 60 samples for quantification of protein succinylation. 60 additional whole liver samples were collected from the same mice, which were used to quantify metabolites. The controls employed were normal chow-fed mice at each feeding timepont (2 weeks or 10 weeks), for a total of 10 control biological replicates. Batch-randomization was used during sample preparation and mass spectrometry data collection, where 1 sample from each group was processed in each of 5 batches, for a total of 12 samples prepared and analyzed in each batch. Given the large number of conditions compared, we chose 5 samples per condition to balance statistical power with total study size.

### Experimental model conditions

All protocols were approved by the IACUC of the Joslin Diabetes Center and were in accordance with NIH guidelines. Mice were housed at 20–22°C on a 12 h light/dark cycle with ad libitum access to food and water. C57Bl6, male mice at 6 weeks of age were purchased from Jackson Laboratory and fed either chow diet (Mouse Diet 9F, PharmaServ) or HFD (Research diets, D12492) for 10 weeks. Caloric composition of chow diet consisted of 23% protein, 21.6% fat and 55.4% carbohydrates, while HFD had 20% protein, 60% fat and 20% carbohydrates. Mice were watered with either tap water or 30% (w/v) fructose or 30% glucose solution in water.

Mice were weighed and their food intake was recorded once per week. Before sacrifice, mice were random-fed. Mice were sacrificed from 8 to 11am, slightly after the start of the light cycle, and one mouse from each cage, i.e. dietary group, was utilized before sacrificing the next mouse in the same cage. This was repeated until all four mice per cage were sacrificed. Mice were euthanized using CO2 followed by cervical dislocation. Liver mitochondria were isolated as described previously [35–37]. Briefly, liver tissue was minced and rinsed with MSHE buffer (210 mM mannitol, 70 mM Sucrose, 5 mM HEPES, 1 mM EGTA, pH 7.4) until free of blood, and then dounce homogenized in MSHE+0.5% weight/volume BSA +0.5 μM trichrostatin A (TSA) and 10 mM nicotinamide. Homogenate was centrifuged at 600 x gravity for 10 minutes at 4°C, filtered through 2 layers of cheesecloth, and then spun at 15,000 x gravity for 10 min at 4°C to pellet mitochondria. Pellets were resuspended in the same buffer three times and centrifuged again at 15,000 x gravity for 10 min at 4°C to pellet mitochondria again.

### Mitochondria protein digestion and acetylated peptide enrichment

Mitochondria protein was quantified using the BCA assay, and mitochondria containing 1 mg of protein were lysed with addition of 100 μL of 8M Urea, 10 μL of 10% maltoside, 10 μL of 1M TEAB buffer (pH 8.5), and water to a final volume of 200 μL. Proteins were reduced for 30 minutes at 37° Celsius by addition of DTT to a final concentration of 4.5 mM. Reduced protein solutions were then cooled to room temperature and alkylated by the addition of iodoacetamide to a final concentration of 10 mM for 30 min at room temperature. The alkylated protein solution was then diluted to 1 mL final volume using 50 mM TEAB, pH 8.5, and trypsin was added at a ratio of 1 part trypsin per 50 parts mitochondrial protein (wt:wt, 20 μg per sample). Trypsin digestion proceeded overnight for 18 hours at 37° C. Digestion was quenched by addition of formic acid to a final concentration of 1%, and insoluble material was precipitated by centrifugation at 1,800 relative centrifugal force for 15 min at room temperature. Soluble peptides were then desalted using Waters’ Oasis HLB Vacuum cartridges (30 mg sorbent, 1 mL volume). Peptides were eluted with 1.2 mL of 80% acetonitrile (ACN) / 0.2% formic acid (FA) /19.8% water, and dried to completion using a vacuum centrifugal concentrator. Desalted peptides were then resuspended in 1.4 mL of IAP buffer (50mM MOPS, 10 mM Na_2_PO_4_, 50 mM NaCl) for immunoprecipitation. Acetylated peptides were enriched using anti-acetyl lysine antibody-bead conjugate (PTMScan #13416, Cell Signaling Technologies) using 10 μL of beads per sample (1/4 of the manufacturer-supplied tube per sample, approximately 62.5 μg of conjugated antibody). Immunoprecipitation proceeded overnight at 4° C with rocking. The following day, supernatant containing unbound peptides was removed and saved for subsequent anti-succinyl-lysine IP. The beads were washed twice with 1 mL of ice-cold IAP buffer, three times with ice-cold water (Burdick and Jackson HPLC-grade), and then eluted using 100 μL of 0.15% trifluoracetic acid (55 μL followed by a second elution using 45 μL). Unbound peptides from the first IP were then used as input for a second IP [38] using the same method but anti-succinyl lysine antibody-bead conjugate (PTMScan #13764, Cell Signaling Technologies). Peptides eluted from the enrichment were desalted directly using C18 reversed phase StageTips (two eighteen-gauge disks per StageTip), and resuspended in 7 μL of 0.2% formic acid for mass spectrometry analysis.

### Nanoflow liquid chromatography—Tandem mass spectrometry

Peptide separations were carried out using mobile phase A consisting of 97.95% water/0.05% FA/2% acetonitrile, and mobile phase B consisting of 98% acetonitrile/1.95% water/0.05% FA. Samples were loaded onto the first of two sequential C18 column chips (75 μm x 15 cm ChromXP C18-CL chip, 3 μm particles, 300 Å) using an Eksigent cHiPLC system for 30 min at a flow of 0.6 μL per min of mobile phase A. Separation was performed over two 75 μm x 15 cm ChromXP C18-CL analytical chip (30 cm total length) using a gradient from 5% to 40% mobile phase B over 80 minutes. The column chip was washed by increasing to 80% B over 5 minutes that was maintained for 8 minutes, followed by a return to 5% mobile phase B over two minutes that was maintained for 25 minutes to re-equilibrate the column. Eluting peptides were directly electrosprayed into a TripleTOF 5600 mass spectrometer (SCIEX) and analyzed by SWATH data-independent acquisition (DIA). Every SWATH cycle consisted of a 250 ms precursor ion scan from 400–1,250 m/z followed by fragmentation of all ions between 400–1,200 m/z using 64 variable width precursor isolation windows. The SWATH window definitions used for enrichments of acetylated peptides were determined based on the frequency of acetylated peptide identifications in each m/z region using previous data-dependent acquisitions for 42 ms each, resulting in a total cycle time of approximately 3 sec. Non-enriched peptides were also analyzed by SWATH to determine protein-level changes. The SWATH window definitions used to collect data for protein-level changes were based on the cross-lab SWATH study [32]. Fragment ion spectra were collected from 100–2,000 m/z.

### Data analysis

Data for protein-level analysis was analyzed using Spectronaut [39] with our in-house spectral library from DDA runs of pooled protein samples. DIA data from acetyl-peptide enrichment was processed using PIQED [34], which can automatically identify and quantify site-level PTMs using only DIA-MS data, using settings described in the original manuscript. PIQED strings together several tools to accomplish this, including mapDIA [40], TPP tools [41], and DIA-Umpire [42]. PIQED analysis used protein-level quantities determined using Spectronaut for correction of site-level changes by protein-level changes. Correction of acylation by protein quantity changes works by dividing the peak areas for each acylation site by the total peak areas measured for the unmodified protein in parallel mass spectrometry experiments using non-acyl-enriched peptides from the same sample. For example, if the area for an acylation site was found as 1, and the area for the protein was 10, then the ‘pseudo-occupancy’ is calculated as 0.1. If the acylation site doesn’t change in treatment #2, but the protein area in that same treatment increased to 100, then the ‘pseudo-occupancy’ for treatment #2 is calculated as 0.01. Additional analysis was carried out using custom scripts written in R [43]. Functional analysis of protein changes was performed using ClueGO within Cytoscape [44,45].

### Metabolite quantification

50 mg of previously frozen liver tissue was homogenized in 1 ml of 50% aqueous ACN, containing 0.3% FA. Liver amino acids, acyl CoAs and organic acids were analyzed using stable isotope dilution techniques. Acyl carnitines were measured by LC-MS/MS as described previously [46].

### Quantification and statistical analyses

Significant differences for protein levels were computed using student’s t-tests within spectronaut, and significant differences between acetylation sites and succinylation sites were computed using a Bayesian model within mapDIA [40] as part of the PIQED software tool and workflow. Metabolite concentrations were compared using multiple unpaired 2-way t-tests and Benjamani-Hochberg multiple testing correction to compute q-values [47]. Significant protein level changes were defined as log2(FC) >0.58 and q-value < 0.01, and significant site-level changes were defined as log2(FC)>1 and q-value < 0.01. On heatmaps, sites not meeting significance are plotted in grey or white. Fischer exact testing was used to determine functional term enrichment analysis within the ClueGO plugin of cytoscape. From each cohort, we collected data for 5 biological replicates, but due to mass spectrometer software errors, we lost data the fifth replicate of the acetylation enrichment from 10-week HFD+G. Among the succinylation replicates, we lost data for the fifth replicates of the succinylation enrichments from: 2-week F, 2-week G, 10-week F, 10-week HFD, and 10-week HFD+G. From the 2-week control succinylation set, we lost data from two samples, leaving 3 replicates for that group. Therefore, all cohorts had at least 3 biological replicates, but most cohorts had 5 replicates.

## Results

### Overview of global diet-induced remodeling of mouse liver proteins and protein acylation

**Fig 1:**
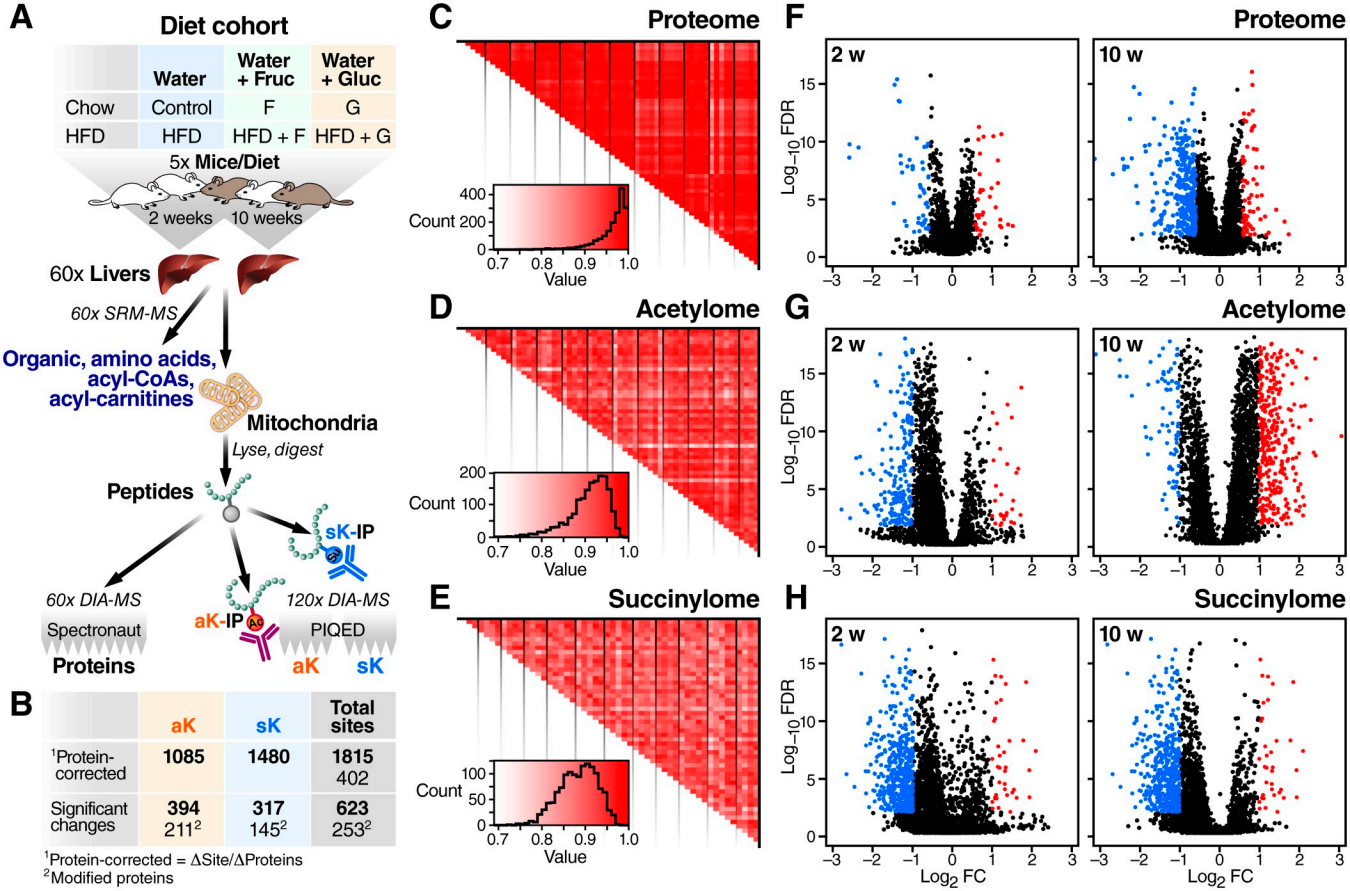
Diet-induced changes in mouse liver proteome, acetylome, and succinylome. **a**, Schematic of the dietary conditions, sample preparation, and analysis workflow. Five mice from each dietary cohort were sacrificed and a portion of liver tissue was used for quantification of organic and amino acids. Mitochondria were also isolated from separated portions of liver tissue and used for measuring alterations in abundance of proteins, protein acetylation, and protein succinylation. **b**, Calculation of protein-normalized acylation and overview of the qualitative and quantitative results. **c-e**, Pearson correlations comparing all peak areas of each biological replicate with all peak areas of other biological replicates, including those from other diet cohorts, for each of the following datasets: proteome **(c)**, acetylome **(d)**, and succinylome **(e)**. The vertical black lines delineate diet groups. **f-h**, Volcano plots showing magnitude and significance of all measurements compared to the 2-week or 10-week control cohort for the proteome **(f)**, acetylome **(g)**, and succinylome **(h)**.

To study how individual macronutrients can contribute to metabolic outcomes, cohorts of mice were fed six different diets. Mice fed SCD or HFD were provided regular water, or water supplemented with 30% fructose or 30% glucose. The diets were administered for 2 or 10 weeks to represent relatively acute and more chronic effects of dietary exposure (Fig 1A). The diet groups are abbreviated as: SCD plus water (control), SCD plus fructose (F), SCD plus glucose (G), HFD plus water (H), HFD plus fructose (HFD+F), and HFD plus glucose (HFD+G). Full metabolic descriptions of the 10-week cohort of mice is available in figure 1 of Softic *et al*. [35]. To summarize metabolic parameters, all diet groups gained significantly more weight than controls, with HFD+F gaining the most weight. Compared to the standard chow diet controls, only the he HFD and HFD+F cohorts had increased blood sugar and insulin, as well as worse glucose tolerance test AUC. Either sugar supplementation alone induced mild hepatic steatosis as assessed by histology, and all HFD groups had greater steatosis as well as significant triglyceride accumulation.

Liver tissue was used to measure a panel of organic and amino acids, as well as acyl CoA and acylcarnitine metabolites by targeted mass spectrometry. In parallel, mitochondria were isolated from liver tissue in order to measure the liver mitochondrial proteome, and protein lysine acetylation and succinylation (Fig 1A). We identified 862 proteins, 1,465 acetylated peptides and 1,957 succinylated peptides (S1, S2 and S3 Tables). Measurement of both protein and lysine modification levels from the same samples allowed correction of acylation changes by changes in protein expression, which was automatically performed by PIQED software [34]. To ensure all sites were corrected for any changes in unmodified protein, the final set of acylation sites was further filtered to only sites where we also quantified the unmodified protein in the parallel standard proteomics experiments. This reduced the final number of protein abundance-corrected acetylation and succinylation sites to 1,085 and 1,480, respectively. Among those sites, 750 lysine residues were found as both acetylated and succinylated (S1 Fig). A total of 1,815 modified lysine residues on 402 proteins (Fig 1B) were identified and quantified.

Quantified proteins and protein acylation sites from each condition showed high reproducibility among all biological replicates, with the average correlation coefficients (Pearson r) of 0.96, 0.91, and 0.88, for proteome, acetylome, and succinylome measurements, respectively (Fig 1C–1E). Our three datasets are therefore of similarly high quality and reproducibility as a recent large-scale phospho-proteomics resource study [48]. We compared each diet to the corresponding 2- or 10-week control group and found that 623 lysine sites in 253 proteins showed at least one statistically-significant diet-induced change (fold change > 2, FDR <0.01, Fig 1B, 1G and 1H). The overall trends in changes in protein abundance, protein acetylation, and protein succinylation are evident from volcano plots of -log_10_(FDR) versus log_2_(FC) (Fig 1F–1H).

### Chronic HFD and glucose induce distinct remodeling of mouse liver mitochondrial proteins

**Fig 2:**
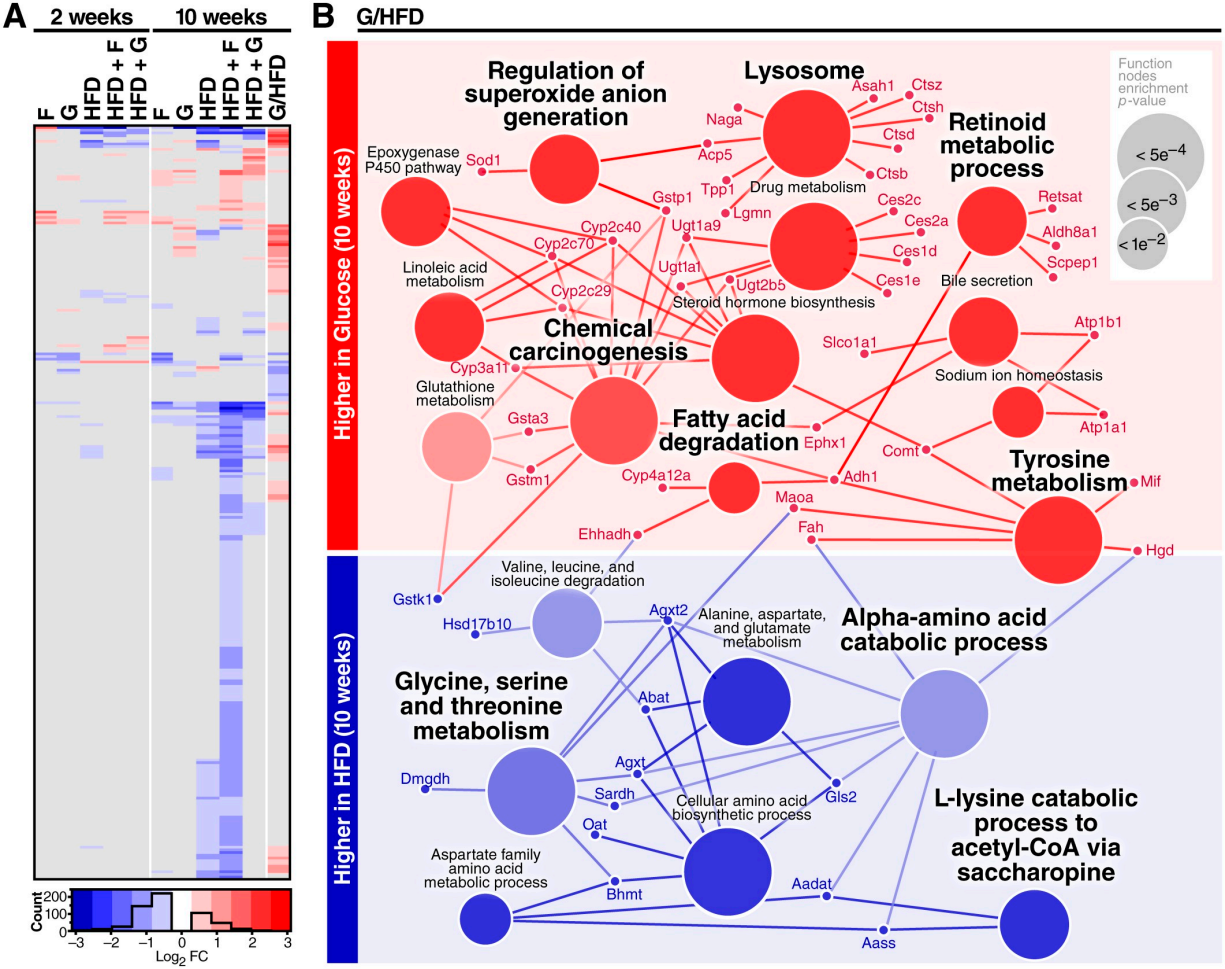
Quantitative protein-level results overview. **a**, Heatmap of 276 proteins that show at least one statistically-significant diet-induced change (q-value < 0.05, fold-change > 1.5). Values in grey are not significant at this threshold. Each diet is compared to the appropriate 2- or 10-week control, except the last column shows the direct comparison of 10-week glucose divided by 10-week HFD. **b**, Proteins showing altered abundance (small circles) between the 10-week glucose cohort and the 10-week HFD cohort in the last column of **(a)** were analyzed for GO and KEGG ontology term enrichment (large circles). Blue indicates lower abundance in 10-week glucose group compared to 10-week HFD group (or higher abundance in the 10-week HFD group than the 10-week Glucose group) and red indicates higher abundance in the 10-week glucose / 10-week HFD comparison (or lower abundance in the 10-week HFD group than the 10-week glucose group).

Automatic correction of acylation site quantification by protein abundance performed by PIQED software was critical in this study due to significant diet-induced mitochondrial proteome abundance changes; 276 proteins significantly changed in at least one comparison (fold change >1.5, q-value < 0.01, Figs 1F and 2A, S4 Table). Most of the protein-level changes resulted from 10 weeks of feeding the three HFDs. Because the liver serves as an important regulator of glucose and lipid homeostasis [49], we also compared the 10-week glucose (G) group with the 10-week HFD group (last column in Fig 2A). Proteins with significantly altered abundance in the 10-week glucose group compared to the 10-week HFD group clearly segregate into separate functional groups (Fig 2B). Mice fed HFD for 10 weeks showed higher abundance of mitochondrial proteins involved in amino acid catabolism than mice fed excess glucose for 10 weeks, whereas mice fed glucose for 10 weeks had increased levels of several proteins involved in lipid metabolism (e.g. Ehhadha and several cytochrome P450s) and cellular redox homeostasis (e.g. Gstm1, Gsta3, Gstp1, and SOD1). The higher relative abundance of redox proteins in liver mitochondria due to excess sugar is consistent with previous reports that sugar metabolism reduces reactive oxygen species (ROS) [50]. Additionally, acid phosphatase 5 (ACP5), which degrades FMN to riboflavin, is lower in HFD, consistent with the increased need for the FAD cofactor in fatty acid oxidation.

Among proteins that increased in the glucose-fed group compared to the HFD group were several enzymes involved in drug metabolism and lipid hormone metabolism, such as cytochrome p450s. Our data suggests that the less deleterious metabolic impact of excess glucose ingestion compared to excess HFD ingestion [35] may be mediated in part by glucose-induced increases in abundance of proteins that perform arachidonic acid hydroxylation (Cyp2c29, Cyp2c40, Cyp2c70), a process that can either activate or inactivate the inflammatory response, and in the case of Cyp2c29, produces an anti-inflammatory epoxyeicosatrienoic acid (EET) [51]. Also, Cyp3a11, a protein that hydroxylates various substrates including testosterone, was higher in glucose-fed animals than HFD-fed animals after 10 weeks. These protein-level changes are consistent with studies that show obesity-related reduction of redox capacity, altered drug metabolism, and increased inflammation in obese mice and humans [52,53].

### Diet-induced changes in amino acids, TCA intermediates, acyl-CoAs, and acyl-carnitines

**Fig 3:**
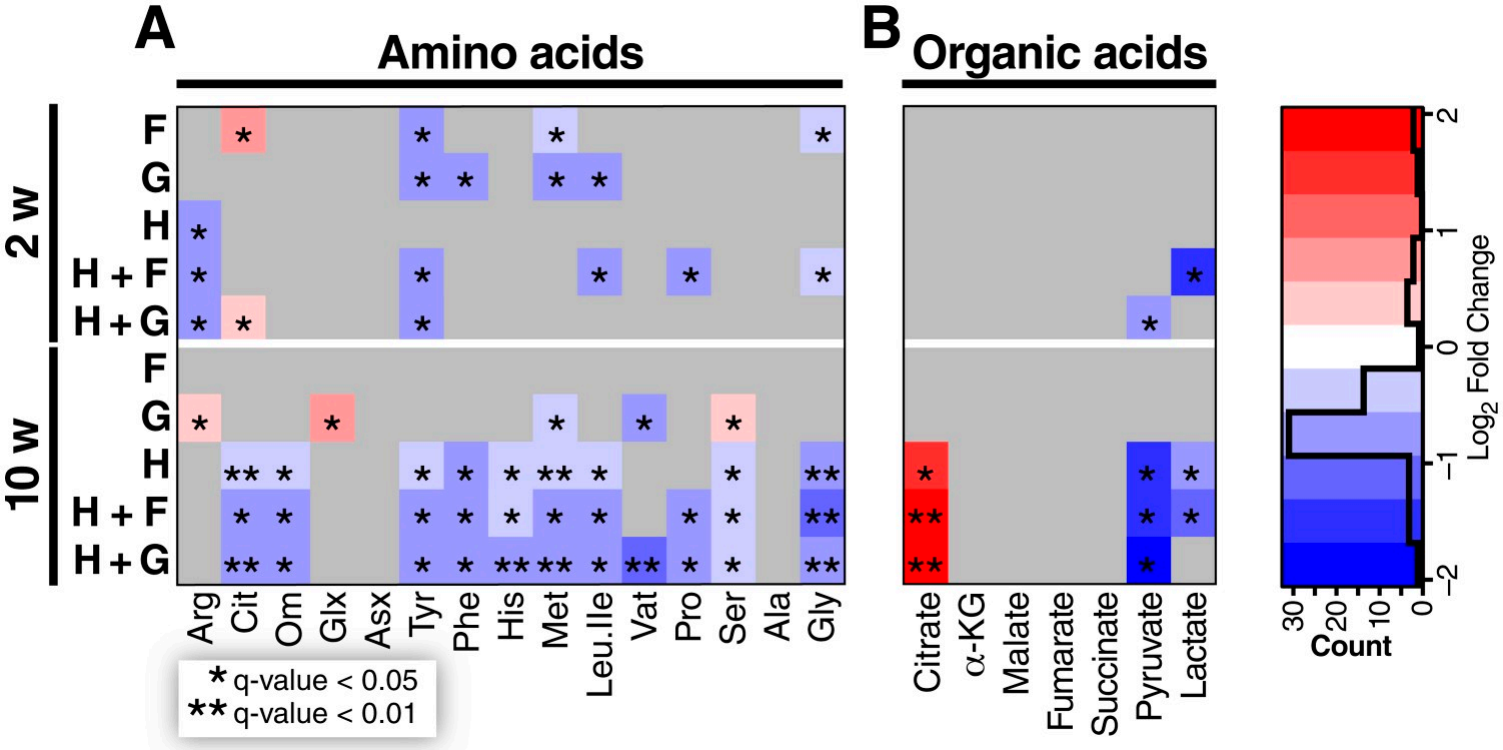
Diet-Induced changes of organic acid and amino acid abundances. Heatmaps show all statistically significant (any fold change, * q-value < 0.05, ** q-value < 0.01) abundance changes in the liver tissue relative to the appropriate 2-week or 10-week control group. **a**, Amino acids and, **b**, organic acids. n = 5 mice.

The dichotomous changes observed in protein abundances due to chronic excess ingestion of sugar compared to chronic excess ingestion of fat are reflected by the metabolite changes observed in livers from the 10-week cohorts. In agreement with the increased levels of several proteins involved in amino acid catabolism, we found a corresponding decrease in multiple amino acids in livers from the HFD groups. Relative to the 10-week control group, mice fed any HFD exhibited significant decreases in glycine, serine, leucine/isoleucine, glutamate/glutamine, methionine, histidine, phenylalanine, tyrosine, and the urea cycle intermediates ornithine and citrulline (Fig 3A). Interestingly, feeding fructose for 10 weeks had no effect on amino acid levels compared to the control group. Unique to the chronic glucose-fed group, levels of arginine, glutamate/glutamine, and serine were increased relative to the 10-week controls. Chronic dietary glucose supplementation was also associated with more modest decreases in methionine and valine. The only other condition where valine was decreased was 10-week HFD+G, suggesting that excess dietary glucose potentiates valine catabolism in the HFD setting. These diet-specific changes were not evident at the 2-week time point, and are thus considered as chronic and not acute responses to the various diets.

Since amino acids can serve as a source of anaplerotic substrates for replenishment of the tricarboxylic acid (TCA) cycle, we also quantified TCA cycle intermediates (Fig 3B). In liver tissue from mice fed HFD, HFD+F, or HFD+G for 10 weeks, citrate was significantly elevated and pyruvate was sharply decreased. Lactate was also lowered in the HFD and HFD+F groups. Citrate is known to inhibit glycolysis in all animals except insects [54], which reinforces the selection of fat as the primary fuel source. This increase in citrate was not evident after 2 weeks of diet feeding, whereas lactate was decreased after 2 weeks of feeding of HFD+F and pyruvate was decreased after 2 weeks of feeding of HFD+G, again suggesting a time-dependent impact of the diets on metabolic fuel selection.

Given the apparent connection between excess dietary fat intake and increased amino acid catabolism, levels of various hepatic acyl-CoA and acyl-carnitines were compared to the respective 2-week or 10-week controls (S2 Fig). Livers from mice fed HFD or HFD+F accumulated various medium and long-chain acyl-CoAs and acyl-carnitines, suggesting high rates of influx of lipids into the β-oxidation pathway and accumulation of incompletely oxidized intermediates. Strikingly, these intermediates did not accumulate in the chronic SCD plus glucose-fed group. Moreover, addition of glucose to HFD almost completely abrogated the accumulation, possibly by increasing lipid metabolism. To be conservative, we cannot rule out the possibility that glucose acts upstream to prevent lipid utilization. Interestingly, acetyl-CoA and succinyl-CoA, which are thought to non-enzymatically induce protein modification, showed minimal alteration among the tested dietary conditions; acetyl-CoA was increased due to acute glucose feeding and decreased due to chronic HFD, while succinyl-CoA was only altered in chronic HFD+G, where it increased. The latter result also supports glucose-related increase in valine metabolism, as valine is metabolized to succinyl-CoA. We also note an increase in triply-unsaturated C10 acyl-CoA and acyl-carnitine that results from all HFDs.

### Opposite changes in protein acetylation and succinylation in response to chronic dietary sugar or fat

**Fig 4:**
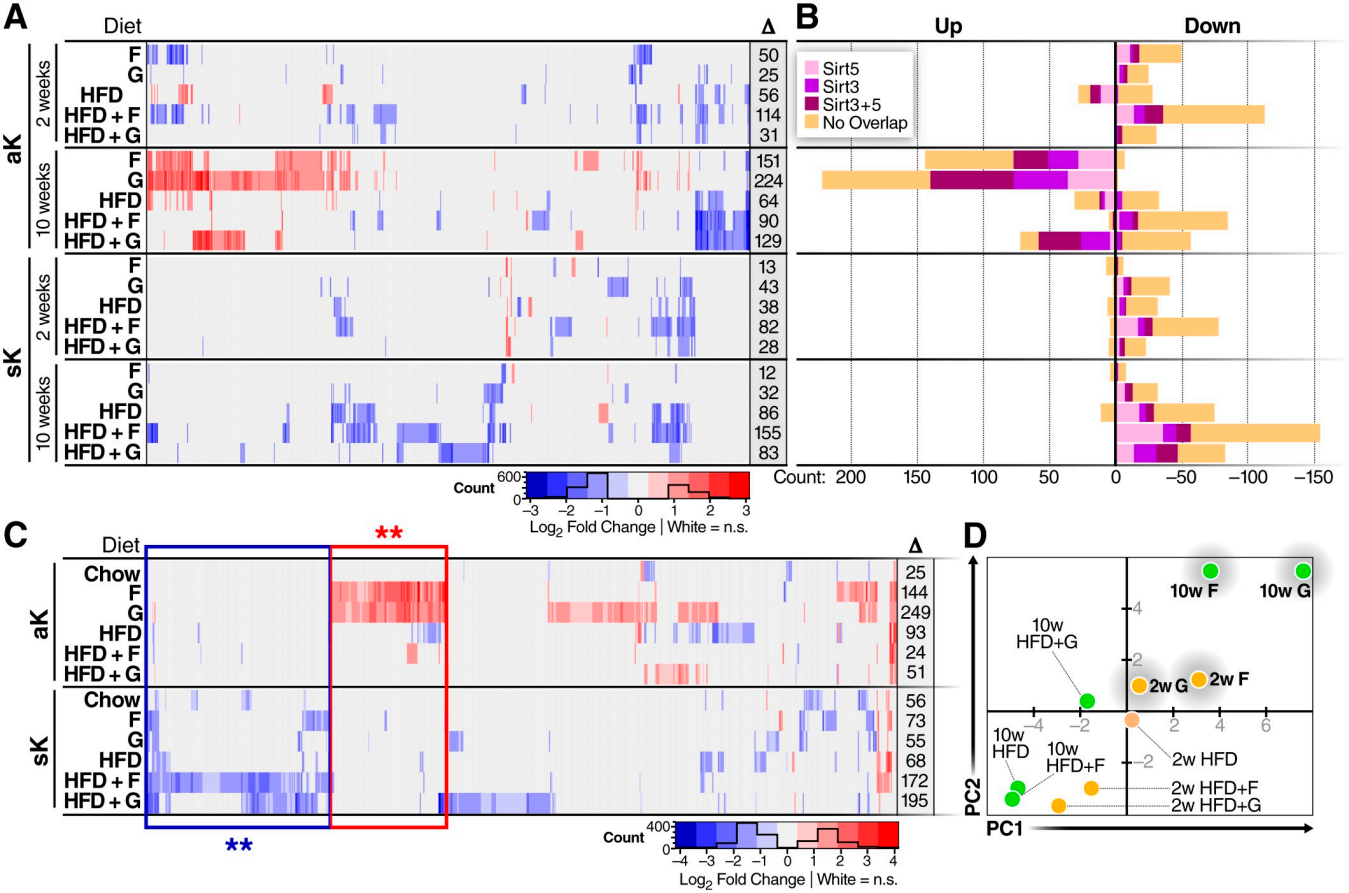
Diet-induced remodeling of liver mitochondria protein acetylation and succinylation. **a**, Heatmap showing 623 modified lysine residues from 253 proteins with at least one statistically-significant change in any diet (FDR<0.01, fold change >2). Columns show acylation sites across the diets, timepoints, and acylation type, and color indicates the fold change of that site relative to the appropriate 2-week or 10-week control. At the right of each row, the total number of significant changes are indicated. **b**, Number of lysine sites showing statistically-significant increase or decrease in abundance of acetylation or succinylation for each diet, and the overlap of those sites with previously-reported Sirt3 or Sirt5-regulated modification sites. **c**, The same as **(a)**, however, these heatmaps illustrate comparisons of 10-week modification abundance over the 2-week abundance. 638 sites in 244 proteins show a time-dependent change due to at least one diet. The numbers at the end of each row indicate the number of significant changes. Groups of boxed sites denote the sets used for functional analysis in Fig 5. **d**, Principle component analysis of the acetylation site levels show segregation of the dietary sugars in chow background from all other groups.

Using the stringently-filtered set of site-level acylation changes relative to respective 2-week or 10-week controls, several general trends become apparent (FDR <0.01, fold-change>2, Fig 4A, S5, S6 and S7 Tables). Both lysine acetylation and succinylation showed more changes after 10 weeks compared to 2 weeks of feeding, and overall acetylation changed more than succinylation. Most of the sites that exhibit decreased acylation due to any diet show less than 50% overlap with previously-described Sirt3- or Sirt5-regulated acylation sites [8,14,55] (Fig 4B). Western blot quantification of Sirt3 and Sirt5 from mice in the 10-week cohort showed no difference in Sirt5, but a slight increase in Sirt3 was observed in mice fed any HFD (S3 Fig). Similar to the divergent changes found in proteins and metabolites, dietary sugar and fat also have dichotomous effects on acetylation and succinylation changes with opposite trends for the two macronutrients for each PTM. Global hyperacetylation was observed in response to chronic glucose or fructose supplementation of chow-fed mice, but chronic HFD was associated with a nearly equal number of increased and decreased sites. Interestingly, addition of glucose to chronic HFD was sufficient to induce some protein hyperacetylation, while added fructose was not. In contrast, chronic feeding of either sugar had almost no effect on protein succinylation, whereas all three of the HFDs induced general hypo-succinylation; the 10-week HFD+F cohort showed the most dramatic effect.

The results also show a clear time dependence of acylation changes. In general, the global trend observed from the 2-week feeding was amplified after 10-weeks of feeding, except for the acute feeding of glucose or fructose. Excess glucose or fructose induced a biphasic response in protein acetylation, with slight decrease in protein acetylation after acute excess but an overall increase after chronic excess. To better understand how acylation adapts from acute to chronic feeding, 10-week acylation was compared to 2-week acylation quantities for each diet (Fig 4C, S8 Table), instead of comparing with the respective 2-week or 10-week control groups as shown in Fig 4A. Not surprisingly, minimal differences were observed comparing the 10-week controls to the 2-week control diets, importantly ruling out maturation or development as causes. Thus, protein acylation changes are dependent on both the type of nutrient excess and the length of exposure. The number of altered acetylation sites found in F, G, and HFD cohorts was similar in the time-dependent analysis (10-week / 2-week, same diet condition) as reported for the diet-dependent analysis (10-week diet /10-week control). However, the time-dependent analysis produced a more modest number of changes in acetylation in the HFD+F and HFD+G cohorts. In contrast, more altered succinylation sites in the F, G, and HFD+G diet groups were found using the time-dependent analysis (10-week / 2-week) compared to the diet-dependent analyses (10-week diet/10-week control). Many of the time-dependent changes were not found in the diet-dependent comparison; by including the time dependent changes, the number of sites with at least one diet-induced change increased by 38% to 861 sites across 301 proteins (panel A in S4 Fig). Notably, in half of the diets, hypo-succinylation was more time-dependent than hyper-acetylation, even though succinyl-CoA is more reactive than acetyl-CoA (Fig 4C) [18]. Together, this analysis of time-dependence indicates that sugar-dependent hyper-acetylation and fat-dependent hypo-succinylation increase with time, with the most significantly altered acetylation resulting from glucose supplementation and the most significantly altered succinylation profile occurring in response to HFD+F feeding.

To further probe the molecular details of diet-induced acylation, we assessed similarity among the dietary groups using both principle component analysis (PCA) and hierarchical clustering (Fig 4D, **panel b in** S4 Fig). The PCA of acetylation changes distinctly segregates the sugar-fed groups from the HFD groups: all groups fed only sugar are in one quadrant and nearly all the HFD-fed groups are in another quadrant. The 10-week HFD+G group is at the opposite end of the PCA plot compared to the 10-week G group. Among the HFD groups, we observed a switch in clustering from 2 to 10 weeks. The 2-week HFDs with added sugar cluster together while HFD without sugar is separate, but the 10-week HFD without sugar clusters closer to HFD+F, while HFD+G is less similar. PCA of diet groups based on succinylation changes also segregates the groups, and the segregation of the 2-week diets mirrored the acetylation changes, but the 10-week diets show sugar-dependent segregation (**panel b in** S4 Fig). Notably, the 10-week HFD, HFD + F, and F groups cluster together. According to the unsupervised hierarchical clustering of diets and acetylation changes, the diets are related primarily by the presence of HFD, and secondarily by the presence of sugar. (**panel c in** S4 Fig). Clustering of succinylation, in contrast, is primarily based on the length of the diet, and secondarily based on the presence of dietary glucose (**panel d in** S4 Fig).

### Diet-induced acylation changes converge at the TCA cycle and amino acid catabolism

**Fig 5:**
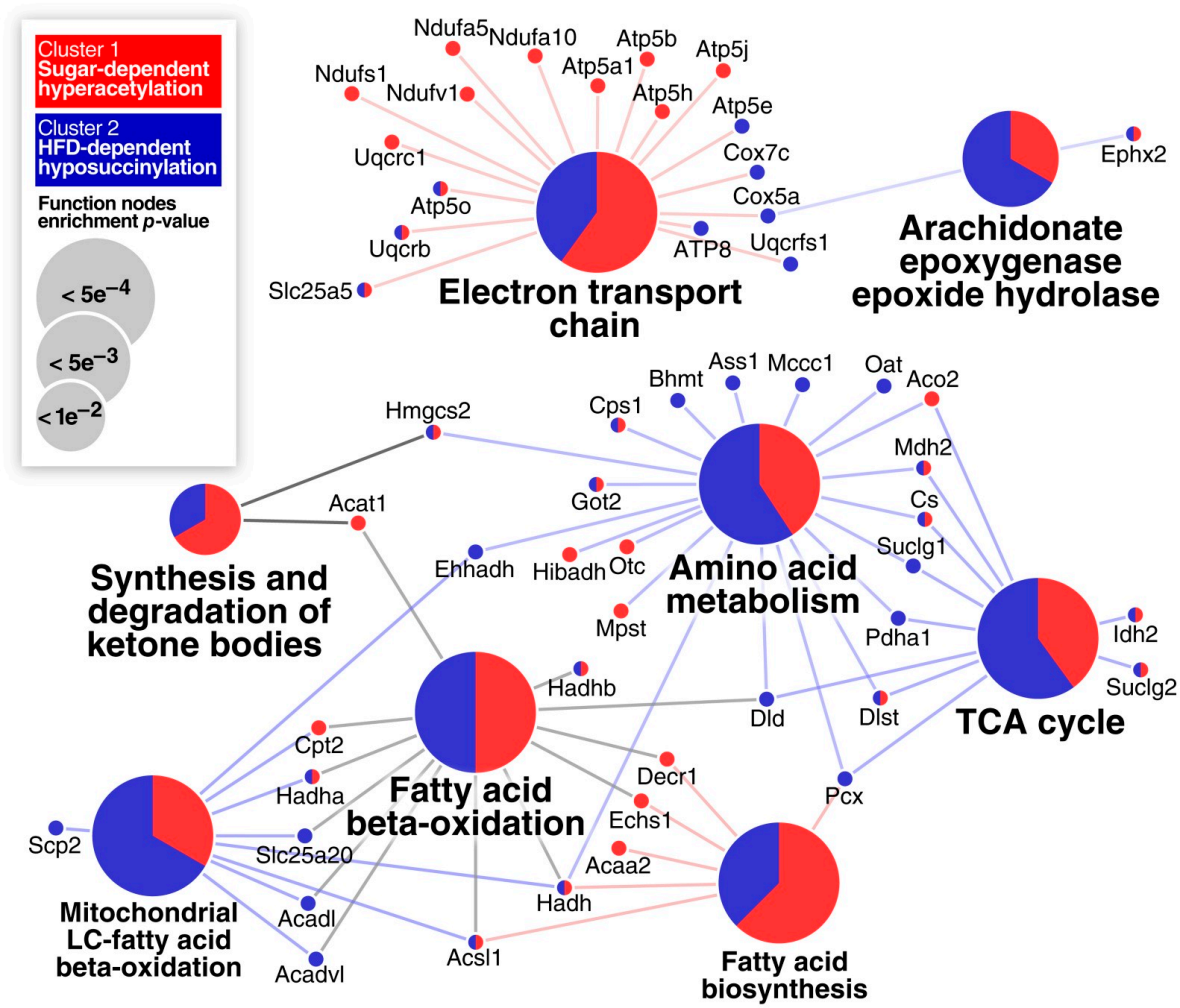
Functional grouping of the proteins showing dichotomous acylation responses. Enriched functional categories of proteins containing sites (denoted by boxes and asterisks in Fig 4C) that undergo site-specific glucose-induced hyper-acetylation (red) or HFD-induced hypo-succinylation (blue). The proportion of red and blue in the functional circles reflects the proportions of proteins from each group that map to this function.

Although distinct clusters of acetylation and succinylation sites are evident from the heatmaps in Fig 4A and 4C, a combined pathway enrichment analysis was done using the proteins containing sites enclosed by boxes marked with double asterisks in Fig 4C (Fig 5). Twelve proteins in the electron transport chain (ETC) and eleven proteins in the combined TCA cycle/amino acid metabolism pathways showed sugar-dependent hyper-acetylation. In contrast, animals fed a HFD showed hypo-succinylation of seventeen proteins involved in amino acid metabolism and the TCA cycle, and eight proteins in the ETC (Fig 5). Several proteins involved in fatty-acid beta-oxidation were also found to be hyper-acetylated in response to sugar feeding and hypo-succinylated by HFDs. Eight proteins in the combined TCA cycle and amino acid metabolism pathways are commonly affected by feeding of sugar and HFD: Cs, Dlst, Idh2, Mdh2, Suclg2, Cps1, Got2, Hmgcs2. The acylation changes on these proteins follow the global trend of hyperacetylation in sugar supplemented diets and hypo-succinylation in HFD (S5 Fig).

**Fig 6:**
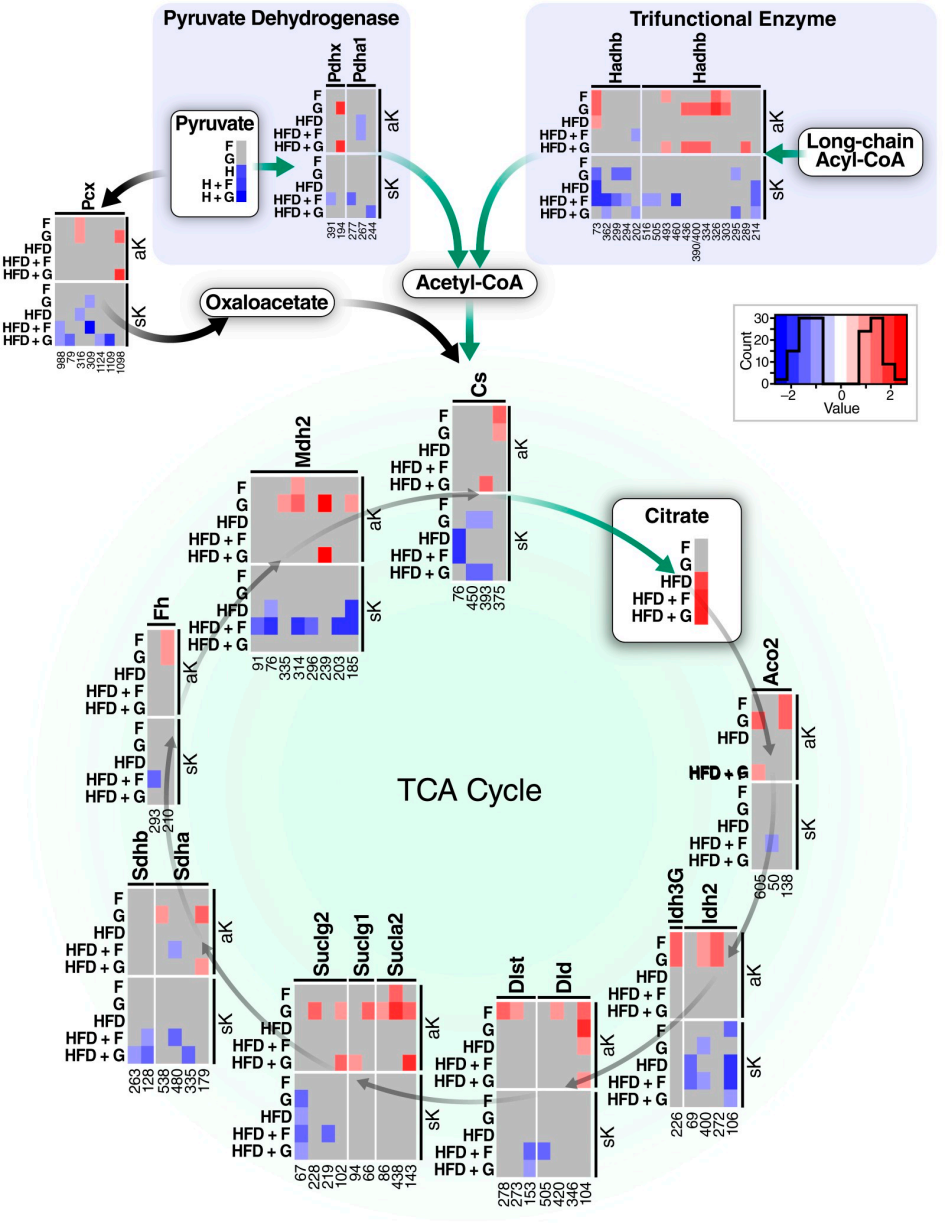
Acetylation and succinylation changes on lysine residues from proteins in the tricarboxylic acid cycle and the proteins that generate acetyl-coA. Schematic of proteins involved in acetyl-CoA generation and the TCA cycle with at least one detected change in acylation after 10 weeks of feeding. Every detected protein in the TCA cycle has at least one glucose-dependent site of hyper-acetylation, and at least one HFD-dependent hypo-succinylation. Pyruvate and citrate quantification from Fig 3 are also shown here.

Given the convergence of fat- and sugar-induced acylation changes on proteins in the TCA cycle, we examined the acetylation and succinylation changes on these proteins across all conditions in more detail (Fig 6). Every protein in the TCA cycle with at least one quantified acetylation change featured glucose-dependent hyper-acetylation, and at least one HFD-dependent hypo-succinylation site. Among the enzymes with this pattern were pyruvate dehydrogenase, which generates acetyl-CoA from sugar, and the trifunctional enzyme, which is involved in generating acetyl CoA from fat. Some enzymes also showed hyperacetylation due to dietary glucose both in SCD and HFD backgrounds, such as K179 of Sdha, K605 of Aco2, K102 of Suclg2, K143 of Sucla2, K194 of Pdhx, several sites in Hadhb, and K239 of MDH2. The latter acetylation site K239 in malate dehydrogenase 2 was found previously to be a strong regulator of Mdh2 activity using acetyl-mimetic mutation K239Q [55]. Taken together, these findings suggest that sugar-dependent hyper-acetylation and HFD-dependent hypo-succinylation of multiple proteins may play a role in the adaptive response to diet, resulting in maximized oxidation of fat, suppression of glucose utilization, and increased amino acid catabolism to provide anaplerotic substrates.

Since all our data suggests functional alteration of amino acid metabolism, acylation of proteins in the amino acid metabolism pathway were compared with those from the TCA cycle. Among the proteins in amino acid metabolism with altered acylation, a total of 116 sites in 27 proteins change due to at least one diet (S5 Fig). The pattern of acylation changes on these proteins nearly follows the overall general trend of sugar-dependent hyper-acetylation and fat-dependent hypo-succinylation, except that ten of these acylation sites showed HFD-dependent hyperacetylation. Notably, six of the ten HFD-dependent hyper-acetylation sites were observed in proteins that regulate ammonia metabolism, including Cps1 (K287, K307, K458), GOT2 (K227, K309), and OTC (K231). This subset of amino acid metabolism proteins has been previously reported to be regulated by acylation [28,56].

## Discussion

In this system-wide, multi-omic study, we report widespread, global protein hyperacetylation resulting from chronic ingestion of dietary sugars, and widespread protein hypo-succinylation resulting from chronic HFD feeding. Accompanying protein-level changes support significant diet-induced remodeling of liver mitochondria metabolism, which was further reflected by altered metabolites. This quantitative analysis of diet-induced proteome, protein acylation, and metabolite remodeling together paints a comprehensive picture of the liver’s response to macronutrient excess.

Our metabolomic and proteomic quantification results reveal complementary aspects of the hepatic response to unique macronutrient stresses. Decreased pyruvate and lactate levels in mice fed HFD are likely explained by the increase in citrate, which is known to inhibit the key glycolytic enzyme PFK1 [57,58]. This increase in citrate levels was unlikely to be driven by differences in citrate synthase activity, which trended higher in all diets relative to controls (S6 Fig). Instead, the citrate accumulation observed in HFD-fed mice relative to glucose-fed mice appears to be linked to increased abundance of proteins involved in amino acid catabolism, a hypothesis also supported by the clear decreases in abundance of nearly all amino acids measured. High levels of plasma citrate have been previously observed in patients with non-alcoholic fatty liver disease, where it was also found to promote the production of ROS [59], possibly due to chelation of ionic iron thereby allowing Fenton chemistry [60]. This is consistent with the decreases in ROS metabolizing/neutralizing enzymes such as SOD1 and glutathione metabolism proteins in mice fed HFD relative to mice fed excess glucose. The proteomic and metabolomic results therefore provide new mechanistic insight into the altered metabolic physiology of obesity.

The most salient finding from the acetylome and succinylome results is the dichotomy of sugar and fat dependent changes. Either sugar induced protein hyper-acetylation, whereas all of the HFD induced protein hypo-succinylation. From our data we cannot exactly determine the mechanism of whether these changes are due to altered ‘on’ rates, sirtuin activity, and/or NAD+ levels. Further, even though we observed low overlap of diet-regulated sites and sirtuin-regulated sites, we do not know if the sites were not regulated in previous studies due to the lack of dietary stress, abundance below the detection limit, or if these diet-regulated sites are truely not regulated by sirtuins. Still, although glucose-dependent hyperacetylation of MDH2 was previously reported in hepatocytes exposed to high concentrations of glucose (Zhao et al., 2010), but our study provides the first evidence of the global nature of protein hyperacetylation in response to excess glucose *in vivo*. Also notably, even though we found significant overlap in acetylation and succinylation sites, we observed very little crosstalk; acetylation sites that change in one diet rarely have alterations of succinylation in the same diet (see Fig 4A and 4C, **and** S1 **and panel a in** S4 Figs). This result is consistent with a non-enzymatic mechanism of mitochondrial protein acylation due to different routes of acetyl-CoA and succinyl-CoA production. These non-enzymatic modifiers may serve to block other forms of lysine modifications, such as ubiquitination and SUMOylation [61].

Since the discovery of mitochondrial protein acylation, several studies have attempted to find site-specific functional effects of acylation using mutagenesis to change lysine into acyl-mimicking residues, such as lysine to glutamine, resulting in altered function. Experiments that introduce mutations to mimic acylation, using either recombinant protein *in vitro* or transfection of cells, reflect a state where all or most of the protein is acylated. However, several recent studies have shown that nearly all protein acylations are present at very low stoichiometry, with estimates of 1 in 1000 molecules acetylated (0.1%) [62–64], raising concerns about the potential functional effects on protein activity. However, if protein acylation is activating, as is the case of tyrosine phosphorylation, then even a low % of modification could have a significant effect, for example by altering protein-protein interactions. Conversely, if protein acylation is inhibitory, the cumulative effects of these small enzyme inhibitions may result in major pathway-level activity changes. For example, if acetylation of ten proteins in a metabolic pathway are each reduced to 99% activity, then the overall flux through this pathway would be reduced to 90%. If instead those proteins were each reduced to 90% activity, then the total pathway activity could be as low as 35%. Thus, our data demonstrating modification of multiple proteins within specific metabolic pathways in response to changes in dietary composition could suggest that even low-level modifications might be contributing to regulation of metabolic fuel selection.

Previous studies reported that chronic HFD induces protein hyperacetylation [26,65,66], which was not observed in the present study. Studies have concluded that acetyl-CoA from fat metabolism is the sole source of mitochondria protein hyperacetylation [25], and that lipids can drive histone acetylation [25,67]. However, Pougovkina *et al*. did not explicitly exclude sugar as the carbon source of hyperacetylation. In fact, other work has shown that carbon from glucose does also provide carbon for protein acetylation [68,69]. Still, there are several experimental differences between our work and previous HFD studies that could account for the apparently disparate observations. For example, compared to at least some experiments reported in previous studies in mice, the present study used a different HFD with less sugar, i.e. ~9% sucrose here, ~29% sucrose in previous studies, as well as a different HFD composition. The second possibility is the different duration of feeding; previous studies administered HFD for at least 13 weeks, compared to this study where mice were fed HFD for only 10 weeks. Given that chronic HFD can induce elevated resting glucose [70] the hyper-acetylation observed from longer-term chronic HFD may actually result from diet-induced hyperglycemia, explaining the time dependence. A third potential explanation is that mice in previous studies were fasted before sacrifice whereas the mice used here were random-fed, and it is possible that acylation patterns can vary in fasted versus fed conditions. Consistent with this idea, lysine acetylation is known to increase in fasting in parallel with increases in circulating free fatty acids, and the fasting-induced increase in acetylation can be blocked by inhibition of lipolysis [71]. This shows that fatty acids can increase acetylation when they are a primary source of acetyl-CoA. Fourth, mice in some previous studies were sacrificed immediately at the start of the light cycle, whereas mice in this study were sacrificed slightly after the start of the light cycle. This could be important because some liver protein acetylation is known to be circadian [72]. Finally, differences in gut microbiota resulting from specific animal facilities are known to have strong impacts on metabolism [73,74]. We cannot explicitly rule out contributions from any of these variables. Nevertheless, under the conditions defined in the current study, we observed a clear dichotomy of effects of HFD and sugar supplementation on protein acetylation and succinylation in animals sacrificed during ad-lib exposure to their respective diets. Further work is needed to dissect the roles of excess sugar and fat in regulating protein acylation, including which carbon source is the preferred protein modifier.

Interpretation of the acyl-CoA and acyl-carnitine measurements in light of the acylation results provides further value to this resource. First, the HFD and HFD+F cohorts had the smallest changes in protein acetylation; however, these groups had the greatest accumulation of long-chain acyl-CoAs (S2 Fig). Given the high concentrations of these numerous RACS, it is possible that mitochondrial protein lysine residues are acylated with longer-chain acyl groups that prevent acetylation and succinylation. Also, free CoA levels are elevated in every 10-week diet except those given excess dietary glucose, and the additional free-CoA may serve as a thiolate sink for acyl groups, again limiting lysine acylation. Finally, although acetyl-CoA and succinyl-CoA are needed for protein modification, their concentrations did not correlate with the observed patterns of protein acetylation or succinylation. Although this lack of correlation may seem problematic, protein acylation is a complex and dynamic process, and our data shows that acyl-CoA quantities alone are not sufficient to predict protein acylation. In fact, recent work has also found a lack of correlation between histone acylation and acetyl-CoA levels in the liver [75].

**Fig 7:**
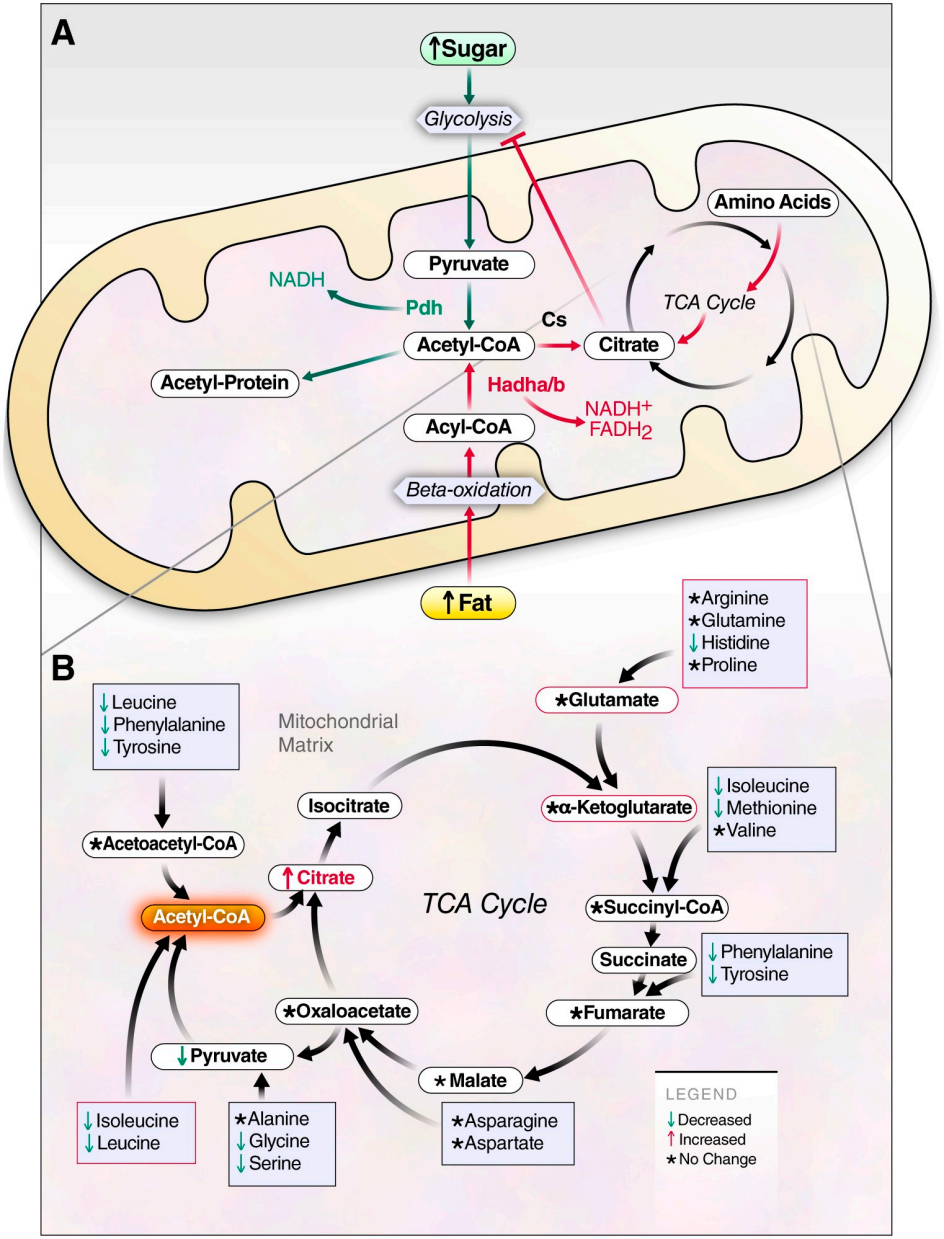
Models of dichotomous forces on mitochondrial functions and protein acylation resulting from excess dietary sugar or fat. **a**, Model describing the central dichotomy of acetyl-CoA production and fate resulting from either glucose or fat, drawn with green or red arrows, respectively. Although both glucose and fat are metabolized through the acetyl-CoA intermediate, acetyl-CoAs are not created equally; acetyl-CoA created from glycolysis results in protein hyperacetylation, but acetyl-CoA produced from fat metabolism does not cause hyperacetylation of protein. HFD is instead associated with citrate accumulation, which is known to inhibit glycolysis. **b**, Model describing the HFD-dependent production of citrate and anaplerosis through amino acid catabolism.

Liver plays a critical role in metabolic regulation by storing glucose as glycogen in the fed state and releasing glucose in the fasted state when lipids become the main fuel source. To achieve this, liver must regulate metabolic fuel selection across the spectrum of lipid, sugar and amino acid substrates, with metabolism of any of these fuels modifying the metabolism of the others [58,76,77]. For example, HFD is known to cause a loss of metabolic flexibility, such that fatty acid oxidation becomes predominant in both the fasted and fed states [77,78], Our comprehensive dataset and analysis supports a dichotomous, nutrient-dependent model of protein acetylation and succinylation, defining a novel set of molecular responses to changes in diet composition that may contribute to modulation of mitochondrial protein function and metabolic fuel selection (Fig 7A). Under our experimental conditions, acetyl-CoA from sugar metabolism uniquely drives mitochondrial protein acetylation. In contrast, in the setting of chronic HF feeding, acetyl-CoA derived from fatty-acid oxidation does not induce protein hyper-acetylation, even after 10 weeks of feeding. Instead HFD is associated with protein hypo-succinylation. HFD feeding also induces citrate accumulation, which is likely generated from fatty acid-derived acetyl CoA condensing with oxaloacetate derived from anaplerotic substrates, including amino acids, as suggested by their reduced levels in livers of HFD mice (Fig 7B). HFD-dependent citrate accumulation is matched by pyruvate reduction, consistent with the classic model of fat-dependent inhibition of glycolysis. The divergent fates of acetyl-CoA and succinyl-CoA reported here may be an important regulatory mechanism contributing to loss of metabolic flexibility in conditions of nutrient overload and obesity.

## Supporting information

S1 Table Details of protein identifications used for quantification. (XLSX)

S2 Table Details of acetylated peptide identifications. (XLSX)

S3 Table Details of succinylated peptide identifications. (XLSX)

S4 Table Quantitative results for proteins. (XLSX)

S5 Table Unfiltered quantitative results for acetylation sites. (XLSX)

S6 Table Unfiltered quantitative results for succinylation sites. (XLSX)

S7 Table Filtered acetylation and succinylation site fold changes matching Fig 4A. (XLSX)

S8 Table Filtered acetylation and succinylation site fold changes matching Fig 4C. (XLSX)

S1 Fig Overlap of protein-corrected succinylation and acetylation sites identified and quantified in this study. (TIF)

S2 Fig Time- and diet-induced remodeling of liver (a) acyl-CoAs and (b) acyl-carnitines. Grey boxes were not significant, colored boxes were statistically significant after two-sided t-test with q-value correction (* q-value < 0.05, ** q-value < 0.01).
(TIF)

S3 Fig Western blot quantification of Sirt3 and Sirt5 from mice in the 10-week cohort. Sirt5 levels were unchanged, but a slight increase in Sirt3 was observed in any HFD-fed group.
(TIF)

S4 Fig Dietary relationships based on changes in protein acetylation and succinylation. **a**, Heatmap showing 861 modified lysine residues from 301 proteins showing at least one statistically significant change in any diet (FDR<0.01, fold change >2). **b**, PCA of diet groups based on changes in succinylation where green dots show the 10w cohorts and yellow dots show the 2 w cohorts. **c**,**d**, unsupervised hierarchical clustering of dietary conditions based on unfiltered changes in acetylation **(b)** and succinylation **(c)**.
(TIF)

S5 Fig Statistically-significant (q-value < 0.01, fold change greater or less than 2) changes in acetylation sites (aK) and succinylation sites (sK) observed for sites in proteins within the amino acid metabolism Wikipathway. (TIF)

S6 Fig Quantification of citrate synthase activity. a, Five replicate mitochondria samples from each 10-week diet were assayed for citrate synthase activity using the kit from Sigma Aldrich. Dichotomous protein acetylation from sugar and fat are not due to a difference in citrate synthase activity, which was higher in samples from all diets compared to the control chow. b, When citrate synthase activity (a) is normalized to the quantification from the proteomic data, the activity becomes more uniform.
(TIF)
