# Supplementary figures and images for: Temporal dynamics of liver mitochondrial protein acetylation and succinylation and metabolites due to high fat diet and/or excess glucose or fructose
Source: PLoS One. 2018 Dec 26;13(12):e0208973. doi: 10.1371/journal.pone.0208973 (PMC6306174; doi:10.1371/journal.pone.0208973)

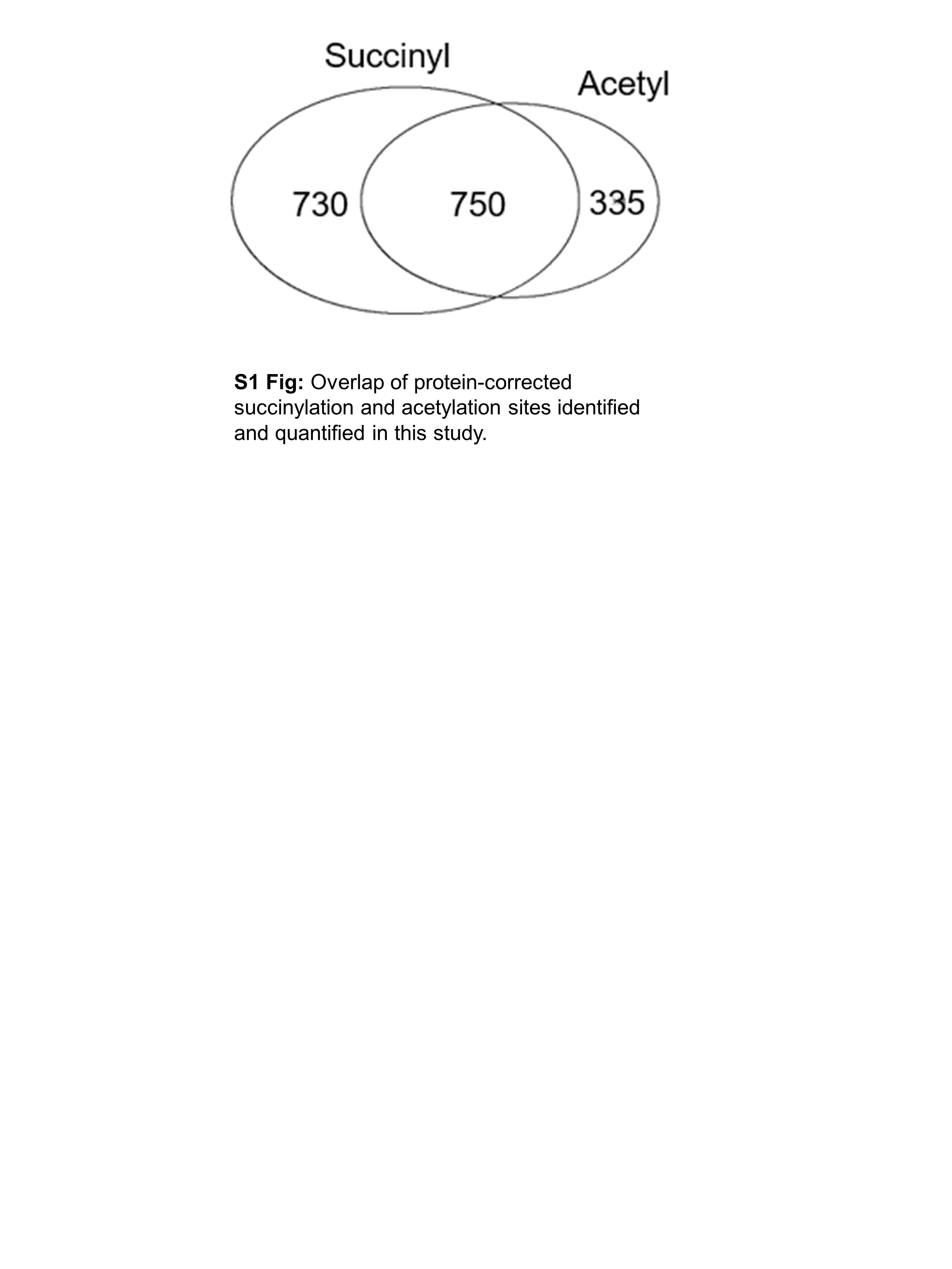

Supplement: S1 Fig — (TIF) [file pone.0208973.s009.tif]

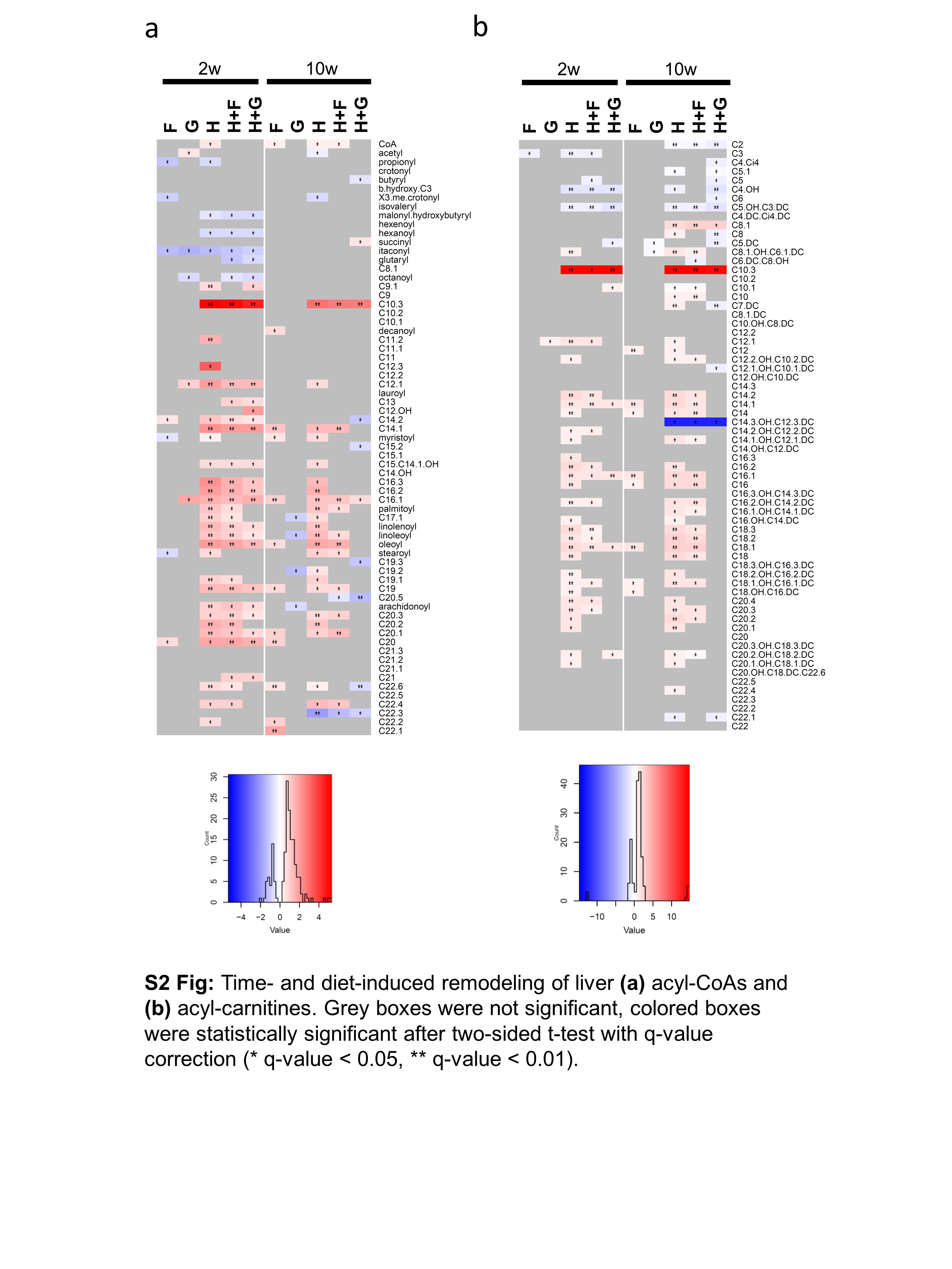

Supplement: S2 Fig — Grey boxes were not significant, colored boxes were statistically significant after two-sided t-test with q-value correction (* q-value < 0.05, ** q-value < 0.01). (TIF) [file pone.0208973.s010.tif]

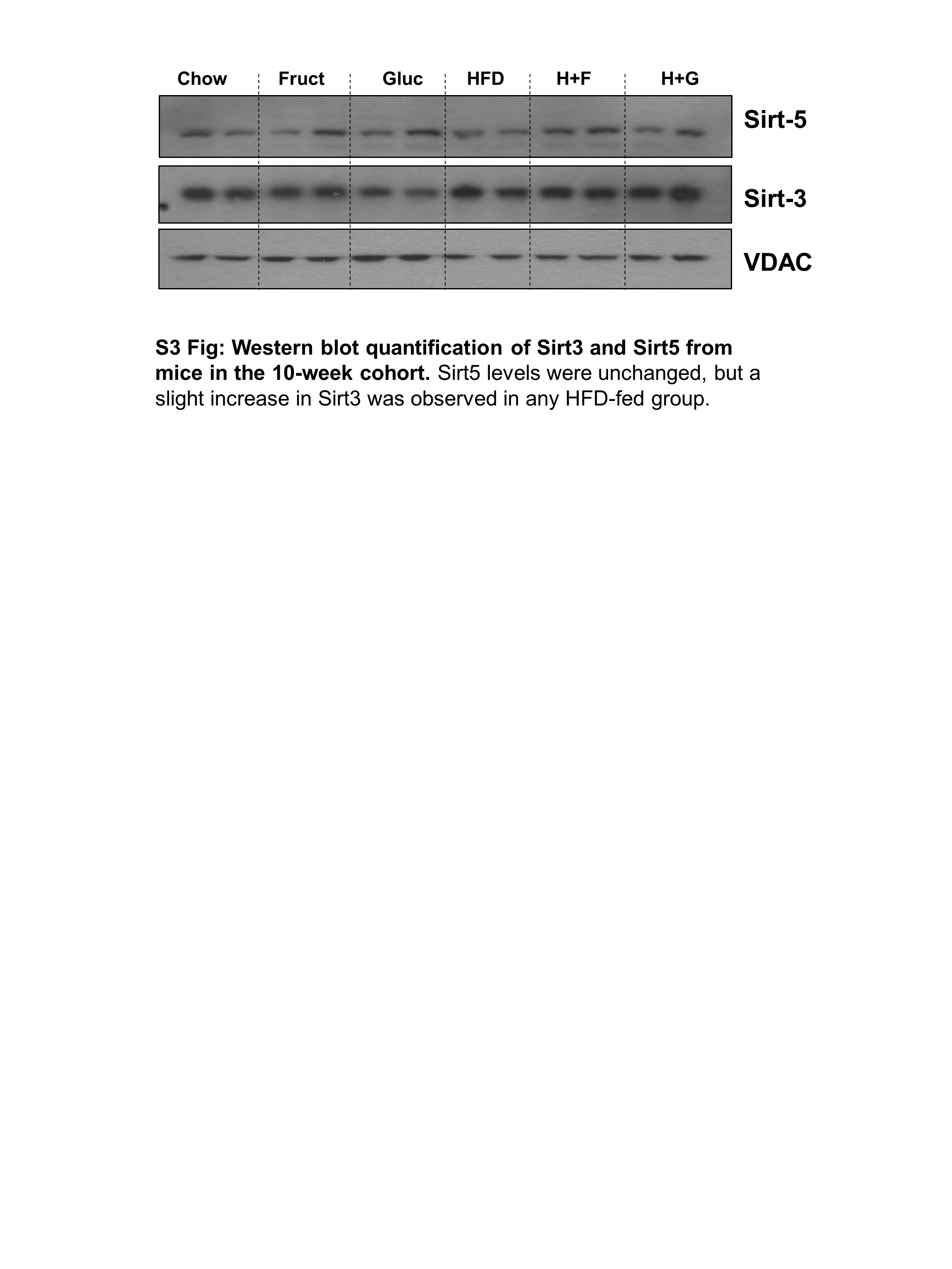

Supplement: S3 Fig — Sirt5 levels were unchanged, but a slight increase in Sirt3 was observed in any HFD-fed group. (TIF) [file pone.0208973.s011.tif]

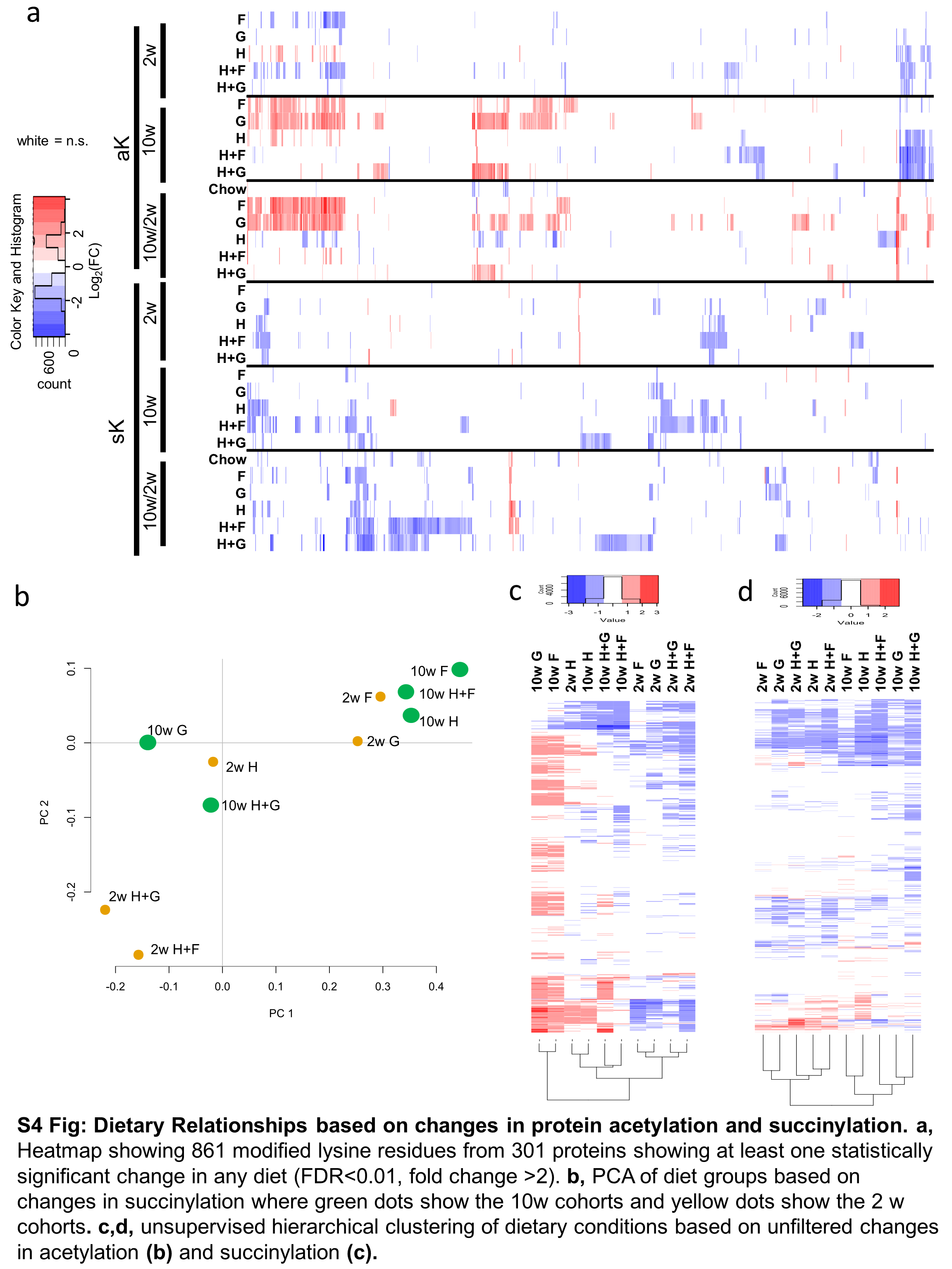

Supplement: S4 Fig — a, Heatmap showing 861 modified lysine residues from 301 proteins showing at least one statistically significant change in any diet (FDR<0.01, fold change >2). b, PCA of diet groups based on changes in succinylation where green dots show the 10w cohorts and yellow dots show the 2 w cohorts. c,d, unsupervised hierarchical clustering of dietary conditions based on unfiltered changes in acetylation (b) and succinylation (c). (TIF) [file pone.0208973.s012.tif]

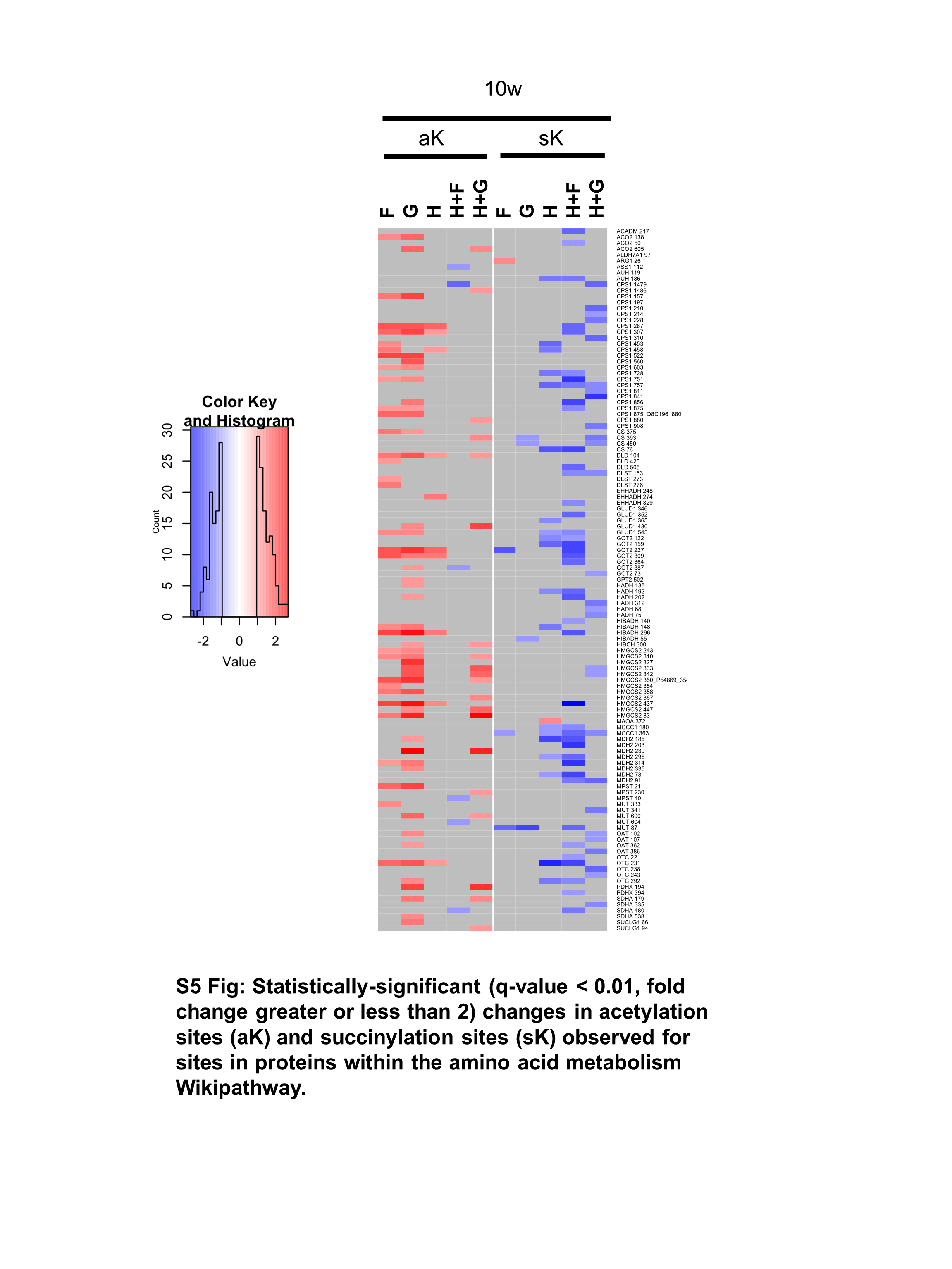

Supplement: S5 Fig — (TIF) [file pone.0208973.s013.tif]

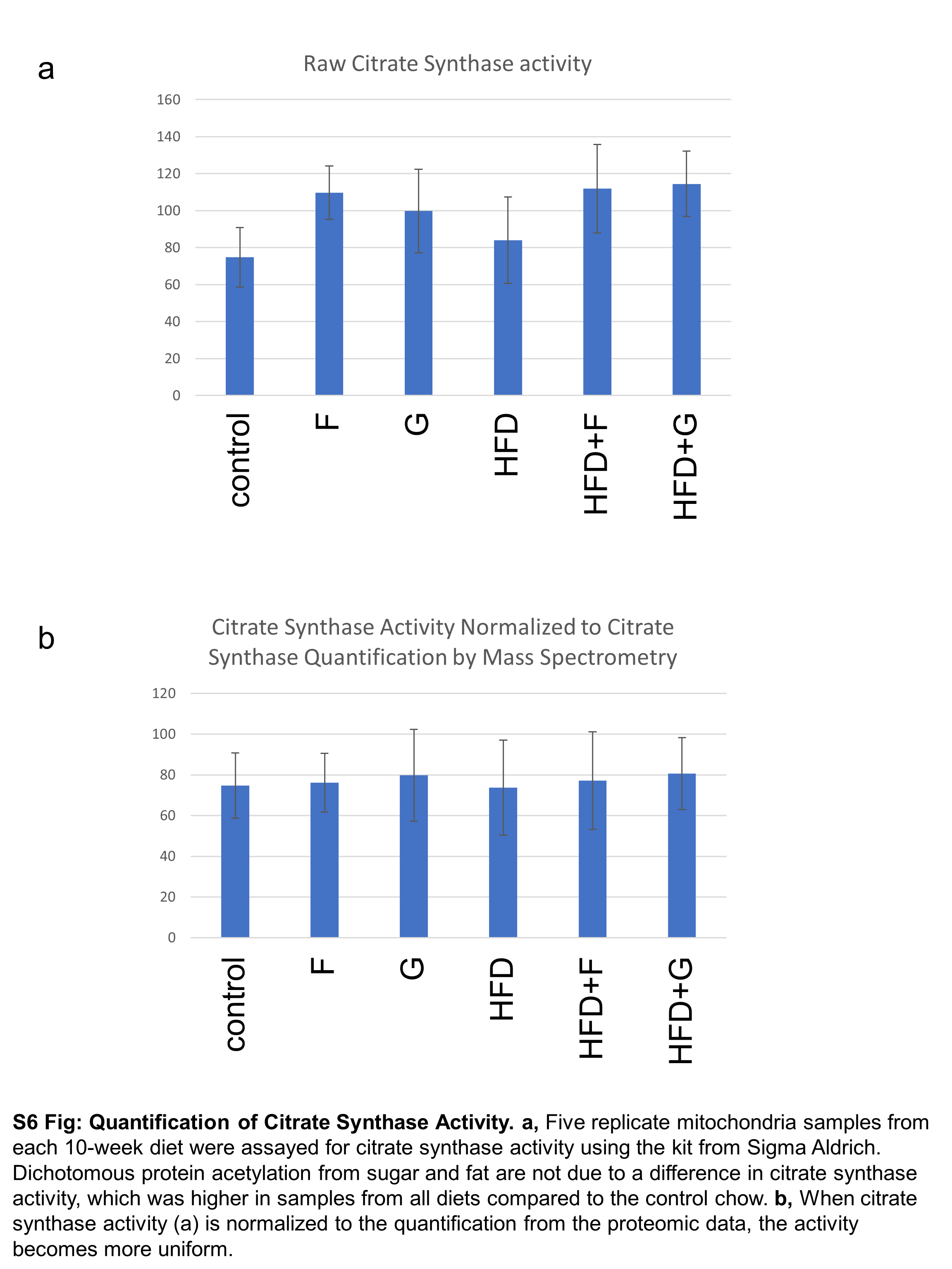

Supplement: S6 Fig — a, Five replicate mitochondria samples from each 10-week diet were assayed for citrate synthase activity using the kit from Sigma Aldrich. Dichotomous protein acetylation from sugar and fat are not due to a difference in citrate synthase activity, which was higher in samples from all diets compared to the control chow. b, When citrate synthase activity (a) is normalized to the quantification from the proteomic data, the activity becomes more uniform. (TIF) [file pone.0208973.s014.tif]
